# Support Mobile Fog Computing Test in piFogBedII

**DOI:** 10.3390/s20071900

**Published:** 2020-03-29

**Authors:** Qiaozhi Xu, Junxing Zhang, Bulganmaa Togookhuu

**Affiliations:** 1 College of Computer Science, Inner Mongolia University, Hohhot 010021, China; ciecxqz@imnu.edu.cn; 2 College of Computer Science and Technology, Inner Mongolia Normal University, Hohhot 010022, China; 3 School of Engineering and Technology, Mongolian University of Life Sciences, Ulaanbaatar 17024, Mongolia; bulganmaa@muls.edu.mn

**Keywords:** fog computing, edge computing, testbed, mobile crowdsourcing, Internet of things, IoT

## Abstract

IoT and 5G technologies are making smart devices, medical devices, cameras and various types of sensors become parts of the Internet, which provides feasibility to the realization of infrastructure and services such as smart homes, smart cities, smart medical technology and smart transportation. Fog computing (edge computing) is a new research field and can accelerate the analysis speed and decision-making for these delay-sensitive applications. It is very important to test functions and performances of various applications and services before they are deployed to the production environment, and current evaluations are more based on various simulation tools; however, the fidelity of the experimental results is a problem for most of network simulation tools. PiFogBed is a fog computing testbed built with real devices, but it does not support the testing of mobile end devices and mobile fog applications. The paper proposes the piFogBedII to support the testing of mobile fog applications by modifying some components in the piFogBed, such as extending the range of end devices, adding the mobile and migration management strategy and inserting a container agent to implement the transparent transmission between end devices and containers. The evaluation results show that it is effective and the delay resulting from the migration strategy and container agent is acceptable.

## 1. Introduction

IoT and 5G technologies are making smart devices, medical devices, cameras and various types of sensors become parts of the Internet [1], which provides feasibility to the realization of infrastructure and services, such as smart homes, smart cities, smart medical technology and smart transportation; and the amount of data they generate is also extremely huge, which challenges the traditional storage and data analysis methods. Cloud computing provides solutions for massive data storage and processing by providing on-demand and scalable resources. However, the high latency is unacceptable for health monitoring, emergency response and other delay-sensitive applications. In addition, it will make the network bandwidth saturated, and the scalability is poor for sending massive datasets to the centralized cloud platform.

Cisco [2] first proposed the concept of fog computing (also known as edge computing) to solve these problems by deploying lightweight computing and storage resources near the end devices, processing and analyzing the data they generated to reduce network congestion, thereby accelerating the analysis speed and decision-making. These lightweight computing resources are called fog. The fog cannot exist independently from the cloud; it offsets and enhances the cloud computing and can be deployed anywhere between end equipment and clouds. As parts of the network, fogs can be routers, switches, servers or mobile stations, and they provide lower delay than cloud computing. Applications such as flow processing, virtual reality/augmented reality, smart traffic control, smart homes and smart cities can benefit from fog computing [3,4,5,6,7,8,9,10].

Fog computing is a new research field, and it is very important to test the functions and performances of various applications and services before they are deployed to the production environment. However, current evaluations are more based on various simulation tools— FogNetSim++ [11], iFogSim [12], EdgeCloudSim [13], etc.—which usually leads to a large difference between the experimental results and the actual situation. A more realistic testing environment can help users find the bottle neck, defect or limitation of their applications. We have designed piFogBed [14] based on raspberry pies, which is a real fog computing testbed that can provide a real fog computing architecture, simulate various network scenarios for users and support users’ testing of real applications. The experimental results obtained on piFogBed have better fidelity than simulators, but, the flexibility and scalability of piFogBed were poor in the following aspects:

(1) The scalability of the end devices is insufficient. In piFogBed, end devices are provided by experimental users, which guarantees their security and privacy, but, for large fog applications such as smart transportation and smart cities, more end equipment is needed, and the self-supply mode will increase users’ costs greatly.

(2) It did not support the testing of mobile applications. The end equipment in piFogBed is connected to a fog node fixedly and cannot move and switch among multiple fog nodes, so the applications can be tested are limited, because there is not a mobility management strategy for the end equipment. But for many fog applications, such as virtual reality, smart transportation and online games, the mobility is a common characteristic.

(3) Users cannot select fog nodes freely. piFogBed automatically selects fog nodes for a user based on the position of his/her end equipment to avoid resource waste, which may lead to a large deviation from users’ actual requirements; in addition, if a user only has a few end devices, he can only apply for fewer fog nodes, which limits the user’s testing tasks.

(4) End devices’ access to a container was not transparent. piFogBed supports running multiple testing tasks on a fog node by using the docker container technology. For a testing task, it may deploy applications on several fog nodes, but the ports mapped to the applications on different fog nodes are not always selfsame, so the user must store each ports on different fog nodes. When an end device logs on a new fog node, it needs to change the connection port, and that increases the user’s workload, especially for mobile applications, which is unacceptable. In addition, it will bring security risks for the internal containers.

To resolve these problems, we modify the piFogBed and propose the piFogBedII to support mobile application testing, expand the scope of end equipment, support a user’s selection of fog node more freely and insert a container agent between external devices and internal containers to make their data transmission transparent, which not only guarantees the security of internal containers, but simplifies users’ work.

The rest of the paper is organized as follows. Section 2 presents related work. Section 3 introduces the new components in piFogBedII. Section 4 presents the implementation of mobile application testing. Section 5 evaluates the effectiveness. The conclusions and future work are presented in Section 6.

## 2. Related Work

Currently, simulation tools are used by many researchers for testing and evaluating fog applications or services because of the low cost and ease of use.

FogNetSim++ is a fog simulator designed based on OMNET++ [15], which provides users with detailed configuration options to simulate a large fog network, and allows researchers to integrate specific mobile models, fog node scheduling algorithms and management handover mechanisms. It is scalable and effective in CPU and memory usage, but does not support virtual machine migration among fog node.

iFogSim is based on CloudSim [16], and it allows users to simulate the fog computing infrastructure and execute simulation applications to test the performance of delay and energy consumption. iFogSim can simulate edge devices, cloud data centers, sensors, network links, data flows and flow processing applications. It integrates simulation services of power monitoring and resource management, and supports cloud deployment and edge deployment of applications. But the location of end devices is static and cannot be updated; on the other hand, it is limited to the discrete event simulators (DES) and has very poor scalability because of the characteristics of CloudSim. Myifogsim [17] extends iFogSim to support mobility by migrating virtual machines between cloudlets. In [18], Naas extends iFogSim to implement data deployment strategy in fog and IoT.

EdgeCloudSim is another fog simulation tool based on CloudSim. It is designed to assess the computing and network requirements of edge computing. EdgeCloudSim supports mobility by providing mobile models, network link models and edge server models. pFogSim [19] extends EdgeCloudSim to include different networks, applications and business process models. IoTSim [20] is also designed to simulate an edge computing environment in which IoT applications send large amounts of data to big data processing systems. As a result, it adds storage and big data processing layers to CloudSim. EdgeCloudSim and IoTSim both inherit the same scalability and DES limitations as iFogSim.

Brogi recently extends the FogTorch [21] and proposes the FogTorchII [22] simulator. It can evaluate the deployment of fog computing infrastructure, and model software functions, hardware functions and QoS attributes, but its scalability is poor.

RECAP [23] supports the modeling for complex and specific requirements to find the best solution for resource management in edge computing systems, but it is difficult to manage and configure, and is not easy to expand or modify.

VirtFogSim [24] is a software toolbox based on matlab, and it is limited to optimizing and tracking the energy and delay performance of task offloading.

Sphere [25] is based on SCORE [26], and can create a cloudlet network based on graph; generate dynamic and parallel workloads; and specify the geographic location, resource density and deployment requirements of workload. But it does not support the mobility of nodes and lacks the migration model of a workload.

Simulation tools simplify the evaluation process, but the differences between the simplified scene and the production environment are very large, especially for the dynamic fog computing scene; the experimental results may be infidelity. Network emulation can solve some problems of simulation to a certain extent and improve the fidelity of experimental results to a certain extent. 

Emufog [27] is an emulation framework designed for fog computing, in which fog nodes are simulated by the maxinet, and applications run in the docker containers. Emufog is more realistic than simulation tools, but it does not support the mobility of clients and fog nodes. 

Fogbed [28] extends the mininet, uses docker containers as virtual nodes and provides the function of building cloud and fog testbeds. It allows users to dynamically add, connect and delete containers in the network topology to simulate the phenomenon of computing instances start and end at any time in the real world cloud and fog environments, but it does not support the management of mobility, security, fault tolerance, scalability and reliability.

Cumulus [29] is a distributed edge computing testbed, but it mainly focuses on task offloading, without considering the whole edge computing system, network topology, network delay and mobility.

Compared with simulation and emulation, the fidelity of an overlay network built with real equipment in a real network is the best. However, it is high cost and difficult to build a real fog computing testbed because the fog equipment produced by different manufacturers is not compatible, and the price is high. We have built the piFogBed using raspberry pies, but it does not support the mobility of end devices. 

Therefore, we modify the piFogBed for supporting the mobility of end devices and name it piFogBedII. We modify piFogBed mainly from four aspects:

1. Adding mobile and migration management for end devices;
2. Expanding the range of end devices;
3. Supporting a user’s selection of fog nodes freely;
4. Inserting a container agent between internal containers and external devices to make their interaction transparent.

## 3. New Components in piFogBedII

### 3.1. Crowd End Devices

Similarly to piFogBed, piFogBedII only provides fog nodes and cloud nodes for users; the difference is that piFogBedII extends the use and scope of end devices in the piFogBed.

Considering the diversity, vulnerability and mobility of end devices, piFogBedII does not provide end devices for users too. Users can either use their own end devices, or employ volunteers and utilize crowdsourcing devices to expand the end equipment. These crowd devices can participate in an experiment as long as they can login on the fog nodes through Wi-Fi and run the end worker app and users’ testing app. As a result, piFogBedII is not only more abundant in the number and types of end devices, but also provides the feasibility for mobile fog application testing.

### 3.2. Logical Components

**Figure 1:**
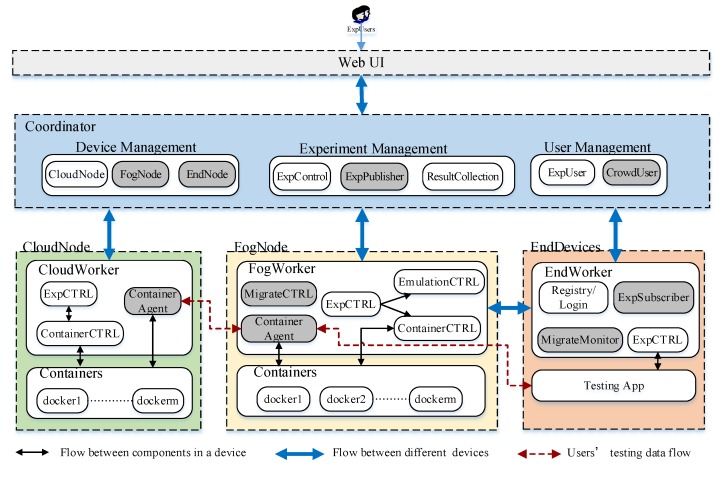
Logical components of piFogBedII.

In piFogBedII, we improve the device management module and allocation method of fog node in the coordinator; moreover, it modifies the way an end device accesses containers and adds mobile management in fog nodes to support mobile fog application testing. The components of piFogBedII are shown in Figure 1, and are the same as those of piFogBed; we still use docker containers to deploy users’ experimental applications on fog nodes and cloud nodes because of certain of their characteristics: open source, light weight, fast startup and small resource consumption. In the following, we mainly introduce the modules which are not included in the piFogBed.

(1) Experiment publisher and experiment subscriber. piFogBedII introduces the crowdsourcing devices into the testbed, so it is important to notify the newest experiment information to crowd users in time. The experiment publisher on the coordinator receives a user’s descriptions about his experiment and publishes the information to some crowd users who are interested in this type experiment to attract them to participating into the experiments. The experiment subscriber runs on crowd end devices, and helps crowd users to subscribe and receive the experiment information published by the experiment publisher that they are interested in.

(2) Container agent. To ensure the transparent access and security of the fog/cloud nodes, we utilize a container agent to isolate the direct interaction of external devices and internal docker containers in fog nodes and cloud nodes. For fog nodes, the container agent receives testing data from end devices or other fog nodes. The testing data flow is produced by the testing app running on the end devices and sent to the container agent running on a fog node; then the container agent transfers the data to a corresponding docker container. When a container running in a fog node wants to send data to another container running in another fog node or cloud node, it should transfer the data to its local container agent, and then the local container agent will connect to the container agent on the destination node and transfer the data to it, so the container agent is the bridge between end devices and internal containers or among containers.

(3) Migration monitor and migration controller. The two modules run on end devices and fog nodes respectively. The migration monitor sends the received signal strength from itself to its current connected fog node; if it finds the signal strength is lower than a threshold, it will send a migration request to another fog node with the largest signal strength.

### 3.3. Experiment Stages

**Figure 2:**
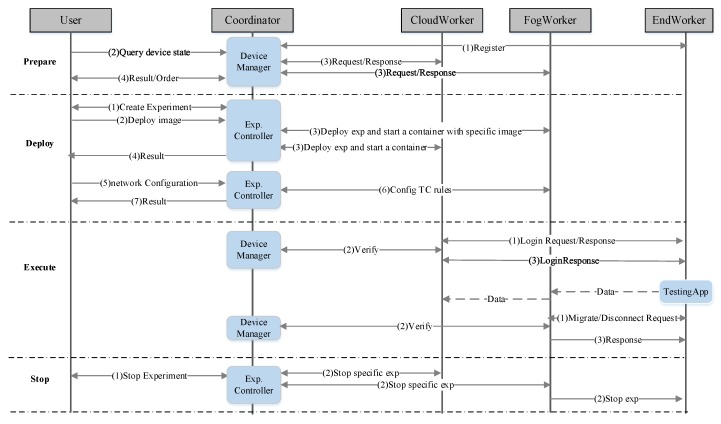
Component interactions in each stage of an experiment.

There are four steps to executing an experiment on the piFogBedII, as shown in Figure 2, and we illustrate them with a simple example. 

Assuming an experimental user develops an edge application to count, in real-time, pedestrian volume passing a road, and he wants to test the function and performance of each part of the application. We suppose that the application consists of three modules: a data aggregate module running on the cloud node, a pedestrian flow collection module running on several fog nodes and a client app running on users’ end devices. As the end device is moved, the client app will connect to the pedestrian flow collection application running on the fog node; each fog node periodically sends the statistical data to the data aggregate module running on the cloud node. Suppose that the user has generated two docker images, which include the data aggregate module and data collection module respectively, and stored them on the Doker Hub, which is the world’s largest library for docker container images. The two images will run on a cloud node and several fog nodes, respectively, later. The user wants to utilize mobile crowdsourcing devices to continuously count the pedestrian volume of a road during a period of time, so he publishes the purpose and demand of the experiment to the piFogBedII platform in advance, which will push the experiment information to users who subscribe to that type of experiment. All end devices participating in the experiment must be installed the endworker, registered on the piFogBedII. Then, the user carries out his experiment according to the following four stages.

#### 3.3.1. Preparation of an Experiment

Firstly, the user views the real-time status of every fog node and cloud node in the test bed through the web, including CPU and memory utilization and the number of experiments running on them currently, and for fog nodes, he can also view their geographical positions. 

Secondly, the user selects appropriate fog nodes according to the experimental requirement. For the above example, the user can select several fog nodes along the road which have enough resources to run the experiment, assuming they are $\left\{f_{1},\;f_{2},\cdots ,f_{m}\right\}$. For cloud nodes, the user gives the number he needs, and then the system allocates the cloud node to him automatically to avoid resource wasting; for example, $\left\{C_{1}\right\}$ in this case. After selection, the user can make a reservation for these devices to avoid them being occupied by other users.

Finally, each end device (including private devices and crowdsourcing devices) participating in the experiment must be installed user’s client app, and connected to one of the fog nodes selected by the user.

#### 3.3.2. Deployment of an Experiment

There are three steps for users to deploy an experiment:

- Create experiment: The user sends a request to the coordinator to create an experiment. The coordinator creates a new experiment in the database for the user and returns the expId to the user.
- Deploy image: The user sends the name of the docker image stored in the docker hub to the coordinator, which will be started on the selected fog nodes (here,$\;\left\{\;f_{1},\;f_{2},\cdots ,f_{m}\right\}$) and cloud nodes (here, $\left\{C_{1}\right\}$). The coordinator sends the image name to the corresponding cloud nodes (here, $\left\{C_{1}\right\}$) and fog nodes (here, $\left\{\;f_{1},\;f_{2},\cdots ,f_{m}\right\}$), and then these nodes download the specified image from the docker hub and startup the container according to the requirement. Let $C_{10}$ and $B_{i}\;\left(i=1,2,\cdots ,m\right)$ denote the containers started on $C_{1}$ and $f_{i}\;\left(i=1,2,\cdots ,m\right)$ respectively for the example.
- Network configuration: After all containers started successfully, the user set the upper containers for containers in the fog nodes. The upper containers may be on another fog node or a cloud node to act for data aggregation. In this example, the upper container of $B_{i}\;\left(i=1,2,\cdots ,m\right)$ in $f_{i}\;\left(i=1,2,\cdots ,m\right)$ is$\;C_{10}$. Secondly, the user sets different delays, bandwidths and losses for different networks link to emulate various Wan scenarios in the LAN environment; for example, the network parameter between $B_{i}\;\left(i=1,2,\cdots ,m\right)$ and $\;C_{10}$ may have delay=0.01, bandwidth=10 MB, loss=0.05 or other values. Then the coordinator sends the topology relationship and emulation parameters to every fog node; for example, telling$\;f_{i}\;$that the upper container of the container $B_{i}$ is $\;C_{10}$ and the emulation parameters between them are {0.01, 10 MB, 0.05}. Finally, each fog node converts these parameters into one or more traffic control rules which can control the net link between a specific docker container running on local and another docker container running on another node. The testing applications are running in the containers; for instance, the aggregate module runs in the container$\;C_{10}\;$and the collection module runs in the container $B_{i}$; then the link between $B_{i}\;$ and $\;C_{10}$ will be shaped by the TC rules.

#### 3.3.3. Execution of an Experiment

Firstly, the participants login their end devices on one of the fog nodes through the endworker. Secondly, they run the client application on their end devices, and the experimental data is sent to the container agent in the fogworker on the fog node $f_{i}$, which will be forwarded to a specific container (such as $B_{i}$ in this example) later. The container (such as $B_{i}$) in the fog node $f_{i}$ transfers the collection data to its upper container ($\;C_{10}$) periodically.

During the progress, some end devices may move and leave the coverage range of the current fog node. The endworker running on end devices monitors the received signal strength to its fog node and sends a migration request to a fog node with the largest signal strength when it finds the current signal strength is lower than a threshold, and completes the migration when it finds the current fog node has switched.

When a participant wants to exit the experiment, he sends an exit request to its fog node through the endworker, and the fog node deletes its information and reports to the coordinator.

At this stage, the experimental user can view current status and relevant information of his experiment through the coordinator.

#### 3.3.4. Termination of an Experiment

The experimental user sends a request to the coordinator if he wants to terminate the experiment, which notifies all fog nodes and cloud nodes in his experiment to close the containers (such as $B_{1},B_{2},\cdots ,B_{m}\;and\;C_{10}$) and release resources. The fog nodes notify the connected end devices and disconnect from them. In addition, the fog nodes remove the traffic control rules relative to the experiment.

## 4. Implementation of Mobile Fog Application Testing

To support mobile fog application testing, piFogBedII needs to solve two problems: (1) migrations of end devices among fogs; (2) transparent data transmission between end devices and docker containers.

### 4.1. Some Key Data Structure

**Figure 3:**
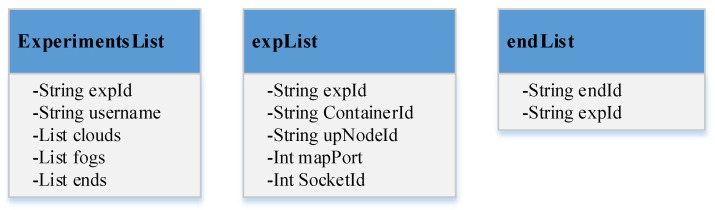
Key data structure used in piFogBedII.

There are three key data structures in the piFogBedII, as shown in Figure 3. The ExperimentsList is maintained by the coordinator, which contains all experiments running on the platform currently. The expList is on every fog node and cloud node, which stores the experiments running on it currently. The endList is on the fog node too, which stores the end devices participating in the experiments running on it.

### 4.2. Migration of End Devices

PiFogBedII extends the end devices, including not only users’ private devices, but also the crowd devices. Crowdsourcing resources enrich the number and types of the end device, and solve the mobility of end devices. However, an end device may move and leave the coverage range of its current fog node, which results in the failure of data transmission. Therefore, the end device must search for a new fog node and complete the migration from the old fog node to the new one, and the function is completed by the migration monitor component in the endworker.

**Figure 4:**
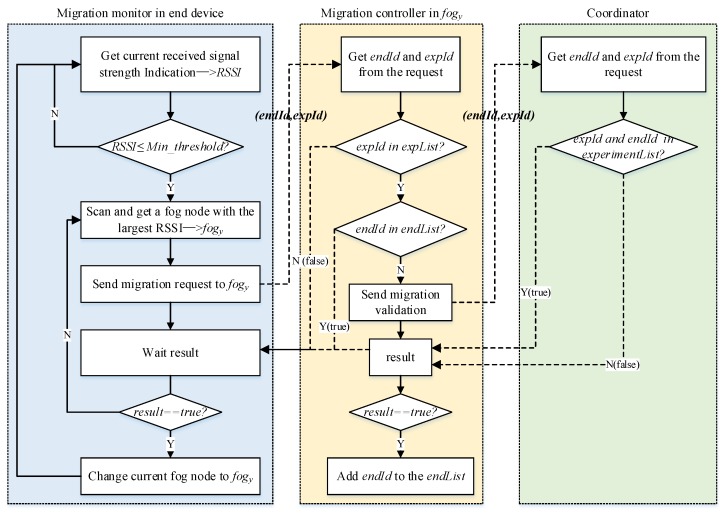
Migration process of an end device.

We implement the migration monitor based on the WifiManager which is a class provided by the android API for the developer to manage all aspects of Wi-Fi connectivity of android devices. The signal will be very weak when the received signal strength indicator (RSSI) is lower than −90 dbm, so we use θ=−85 to denote the migration threshold; i.e., the end device will start to seek a new fog node with the largest RSSI as the migration target if its current RSSI≤−85. The whole migration progress is shown in Figure 4, which is divided into five steps:

1. The migration monitor periodically detects the signal strength from itself to the current fog node ($\;fog_{x}$). If the signal strength is lower than the migration threshold θ, it searches a fog node with the strongest signal strength as its target fog node ($\;fog_{y}$).
2. The migration monitor sends a migration request to the target, and the request data contains the *endId* and *experimentId* which are the unique identifiers of the end device and the experiment of the device participated in at that time.
3. $fog_{y}$ searches the *experimentId* in the *expList*. If the result is null, the $fog_{y}$ refuses the request because the experiment does not run on the fog node and it cannot provide service for the end device; otherwise, it searches the *endId* in the *endList*, and if the result is not null, that indicates the end device has logged in and participated in the experiment on $fog_{y}$ some time ago, so the $fog_{y}$ accepts the migration request directly. If the result is null, $fog_{y}$ validates the end device to the coordinator.
4. If the result returned from the coordinator is successful, $fog_{y}$ sends an acceptance response to the end device; meanwhile, it adds *endId* into its *endList*; otherwise, it sends a rejection response to the end device.
5. The end device modifies $fog_{y}\;$as its new fog node after it receives the acceptance response.
6. The migration request from end devices is processed by the migration controller on the fog node, and we use the reliable transmission technology based on socket to implement the data transmission between them.

### 4.3. Implementation of Transparent Access

Container technology provides a resource isolation environment for multiple applications running on a host at the same time, so that the applications in different containers do not affect each other. In general, a container should open one port at least for data transmission, which will be mapped to a port outside the container. When end devices migrate among different fog nodes, there exists the problem that the same applications in different containers and different fogs are not always mapped to stationary external ports, which not only increases users’ workloads and reduces their experiences, but also exposes the internal containers to outside, which may bring security risks and threats to the containers and fog nodes.

**Figure 5:**
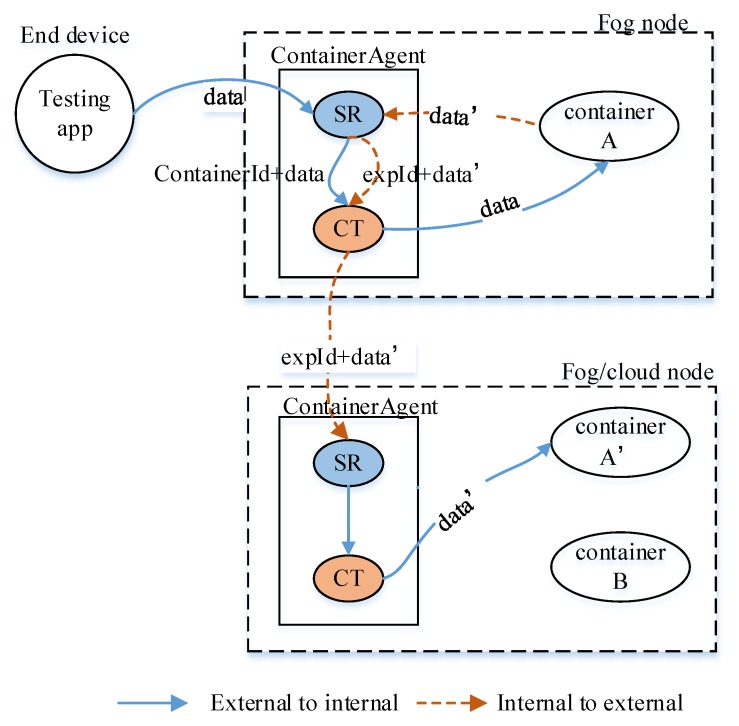
Functions of container agent.

To solve the problem, we design and implement a container agent in the fog node and cloud node to enable the transparent data transmission between external devices and internal containers, as shown in Figure 5. The communications between other devices and container agent are implemented using socket technology. There are two components in the container agent; the SR is a server and the CT is a client. The SR listens at a port and processes requests from other devices or internal containers, and then invokes the CT to send data to the destination. For the data from end devices, the SR obtains the *experimentId* and the *containerId* corresponding to the *endId* of the end devices by searching *endList* and *expList* (in Figure 3), then sends (*containerId, data*) to the CT. For the data from internal containers (for example container *A* in the Figure 5), the SR obtains the *experimentId* corresponding to the *containerId* through searching *expList*, then sends (*experimentId, data*) to the CT. The CT sends the data to a internal container if it receives the *containerId*; otherwise, it sends the data to another container agent running on the upper node of the current node. The CT maintains a long socket connection to every internal container running on the local fog node to save the time of establishing connection with the internal containers and reduce the impact on the transmission delay.

### 4.4. Crowdsourcing Devices

Crowdsourcing [30,31,32] is an effective way to solve difficult tasks, and has been widely used in many fields. With the popularity of mobile devices equipped with various built-in sensors, mobile crowdsourcing has become an important branch of the crowdsourcing field. We introduce the mobile crowdsourcing into the piFogBedII to enrich the end devices, and solve the problems of mobility, high cost, and limited numbers and types of end devices.

A typical mobile crowdsourcing system is composed of a task publisher, a platform and many mobile users (workers). The platform publishes publishers’ tasks to mobile users, and then mobile users select and complete a task using their mobile devices. In piFogBedII, experimental users are task publishers, and end devices are task workers, and the experiment publisher module publishes experimental tasks to crowd mobile devices.

Mobile crowdsourcing allows mobile users to perform tasks that are in their interests and on their behalf. Experimental users describe their experimental tasks through four attributes to enable mobile users to understand and participate in an experiment according to their interests and plans. We assume:

$E_{i}$: Experiment *i* that requiring crowdsourcing resources.

Four tuples {T,D,R,I}: 4 attributes of an experiment, and:

T={collection, computing}: the type of the experiment, data collection or computing;

D={short, long}: the execution duration of the experiment, short or long;

R={high, medium, low}: the resource occupation of the experiment for the end device;

I={high, medium, low}: the reward for participating in the experiment.

**Figure 6:**
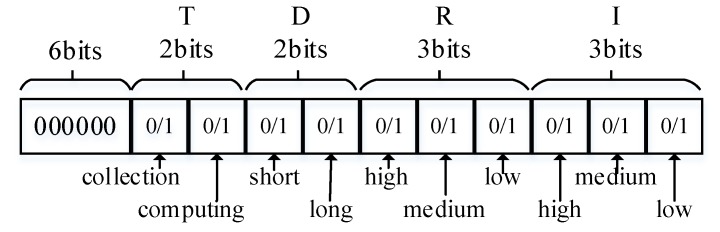
Values of each attribute.

These attributes are all descriptive characteristics, so we digitize these attributes to make convenient the calculation the type of an experiment, as shown in the Figure 6, and for each attribute, only one position can be set as 1.

For an experiment $E_{i}$ and its attributes submitted by the experimental user, we use Equation (1) to calculate its type, and it will be a positive integer of 2 bytes. In the equation, j represents the bit position and $v_{j}$ represents the value of that position. For example, if the attributes tuple is set as {collection, short, low, medium}, then the experiment type will be 1290.

(1) $$V_{e_{i}}=\sum_{j=15}^{0}2^{j}v_{j}$$

**Figure 7:**
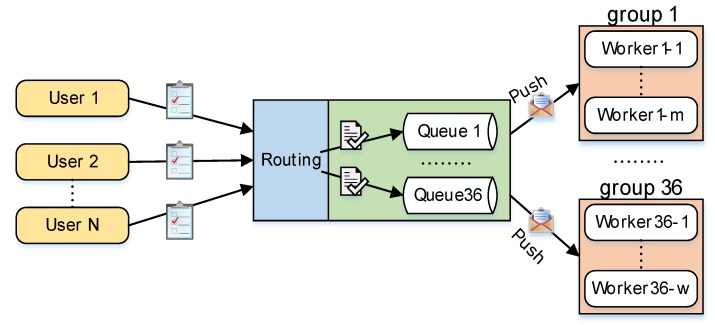
Publish–subscribe model based on active push.

We adopt the publish/subscribe model to enable crowdsourcing users to receive the interested tasks in time, as shown in Figure 7. According to the attributes of an experimental task, crowdsourcing users subscribe to the types attracted him. There are 2×2×3×3 types, so there are 36 queues and user groups. The experiment publisher routes the experiment information to the respective queue and push them to all crowdsourcing end devices in the group according to the experiment type value.

We use MQTT protocol to realize message pushing. MQTT is a publish/subscribe, simple and lightweight messaging protocol, designed for constrained devices and low-bandwidth, high-latency or unreliable networks [33]. MQTT provides one-to-many message publishing based on the Transmission Control Protocol (TCP). The implementation of MQTT requires the communication between the client and server, and in the process of communication, there are three roles: publisher, broker (server) and subscriber. Among them, the publisher and subscriber are both clients, the message broker is the server and the message publisher can be the subscriber at the same time. The messages transmitted by MQTT are divided into two parts: topic and payload. Topic is the type of a message; the subscribers will receive the message content (payload) of a topic after they subscribe, and in piFogBedII, there are 36 topics corresponding to 36 queues of Figure 7; payload is the content of a message, and refers to the specific content to be used by subscribers; here, the experimental description is the payload. MQTT builds the underlying network transmission and establishes connections for clients and the server, and provides them with orderly, lossless, bidirectional transmission.

In piFogBedII, we build the MQTT server based on Apollo which is a messaging broker built from ActiveMQ [34]. The experiment publisher in the coordinator parses the experiment requirements and calculates their type value, and then as the publisher, publishes them to the corresponding topic on the Apollo broker; end devices, as the subscribers, receive the experimental information of related topics pushed by the Apollo broker later.

## 5. Evaluation

We have verified the availability and fidelity of piFogBed in [14]. We will evaluate the performances of the migration strategy and container agent in piFogBedII.

### 5.1. Migration Strategy

**Figure 8:**
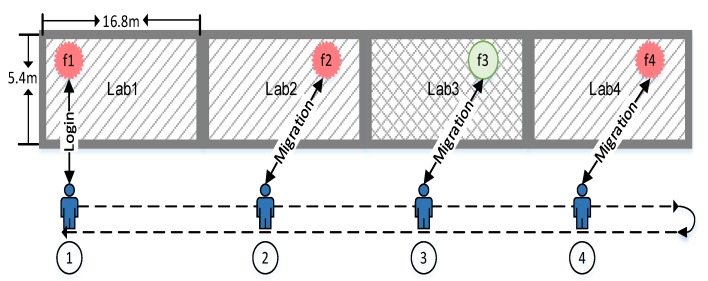
Mobile route of the end device.

In the experiment, we test the migration delay. The experimental deployment is shown in Figure 8. We deploy four raspberry pies in four laboratories as fog nodes. Among them, f1, f2 and f4 run the experiment, while f3 in Lab 3 does not run the experiment. A student holds a smart phone and logs on the f1 from Position 1, and then slowly walks along the corridor to Position 4 and then returns to Position 1. The phone sends migration requests at Positions 2, 3 and 4 respectively, and we test the time from sending request to receiving feedback in each location, and the results are shown in Figure 8.

**Figure 9:**
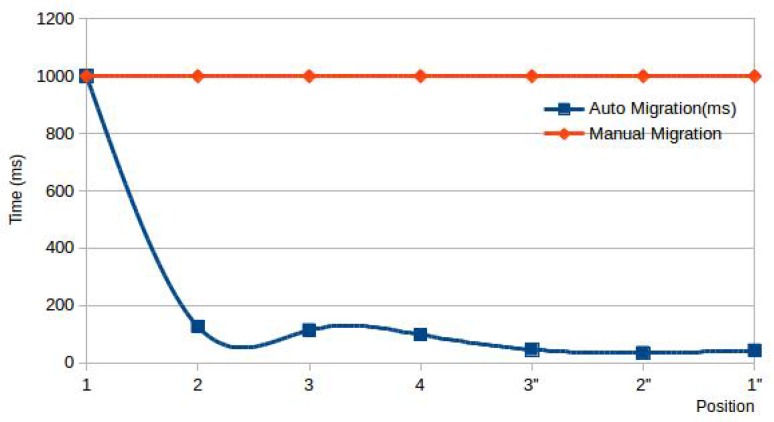
Migration time at different positions.

We test the time spent with no migration strategy used and fog switched manually, mostly over 1000 ms, and we use 1000 ms to represent them in Figure 9. When the student moves from Position 1 to Positions 2 and 4, it will take longer time than from Position 4 to Position 1, because the device is the first to connect them at those positions, and that needs to be verified to the coordinator. In addition, the time spent is shorter at Position 3, because f3 does not run the experiment and directly rejects the request. For the first connection at Position 1, the student needs to log in manually, so it takes a long time.

The experiment proves that the migration process is almost transparent to the user. As long as the experimental user selects appropriate fog nodes, and sets distances between them in appropriate ranges, the mobility of end devices between different fog nodes has little impact on its data transmission, which provides the basis for supporting the mobile fog application testing.

### 5.2. Performance of Container Agent

**Figure 10:**
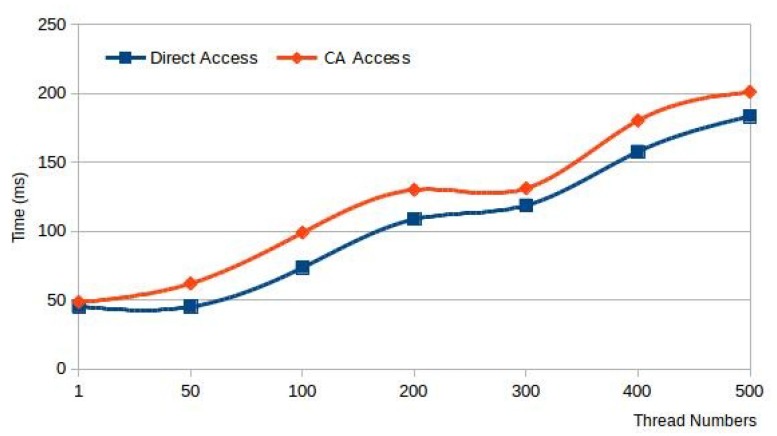
Compare of access directly and by container agent on delay time under different thread numbers.

We evaluate the impact of the container agent on the delay through this experiment. Firstly, we start a container on a fog node and run a server program in the container which feeds back the current time to the user. Then we run a multithreaded client program on a PC to simulate multiple end devices to request the current time from the server program. We test the time spent by the client from sending the request to receiving the feedback under different numbers of threads in the cases of direct access and indirect access with a container agent, and the results are shown in the Figure 10. We find that using the container agent to forward data increases the waiting time from the minimum 3.3 ms to the maximum 25.4 ms, but we think that is acceptable for users.

**Figure 11:**
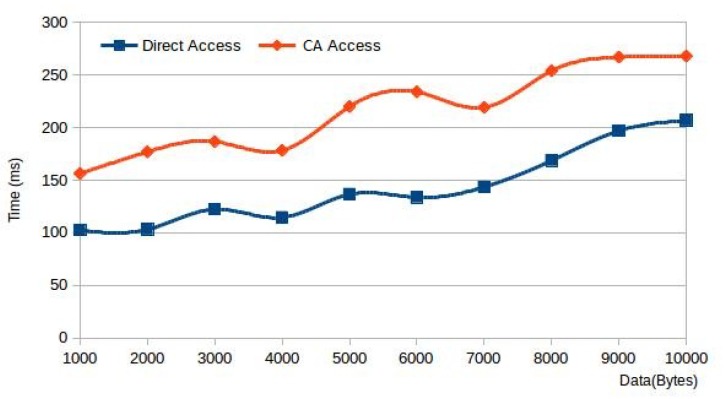
Comparison of access directly and via container agent on delay time under different data amounts.

Finally, we test the impact of the container agent on the user’s waiting time under different transmission data amounts. We use 10 threads to simulate 10 end devices sending different sizes of data to the server program at the same time. We test the time spent from the data sent by the client to receiving response under the conditions of access directly and via the container agent, respectively. The results are as shown in the Figure 11. It can be seen that when there is a large amount of data, the container agent needs to receive data and forward it to the server in the container and it will take a certain time. In our test, the minimum is about 50 ms, and the maximum is about 100 ms. However, the container agent provides users with transparent access to the container; otherwise, users need to connect manually, which takes time longer. We will study more efficient mechanisms to shorten the time of the container agent.

## 6. Conclusions

Fog computing (edge computing) is a new research field and the fog computing testbed plays an important role in promoting the integration of cloud and IoT technology and accelerating the development of fog application. piFogBedII provides more feasibility for mobile fog application testing than piFogBed, and in the future, we will further enhance the performance of the testbed.
